# Stem/progenitor cell marker expression in clear cell renal cell carcinoma: a potential relationship with the immune microenvironment to be explored

**DOI:** 10.1186/s12885-020-06733-4

**Published:** 2020-04-03

**Authors:** Ju-Yoon Yoon, Craig Gedye, Joshua Paterson, Laurie Ailles

**Affiliations:** 1 Department of Laboratory Medicine and Pathobiology, University of Toronto, 27 King’s College Circle, Toronto, Ontario M5S 1A1 Canada; 2 Hunter Medical Research Institute, Newcastle, New South Wales Australia; 3 Department of Medical Biophysics, University of Toronto, Toronto, Ontario Canada

**Keywords:** Clear cell renal cell carcinoma, Stem/progenitor cell, Tumour immune microenvironment, Immunohistochemistry, Tumour microenvironment

## Abstract

**Background:**

Clear cell renal cell carcinoma (ccRCC) is a markedly heterogeneous disease in many aspects, including the tumour microenvironment. Our previous study showed the importance of the tumour microenvironment in ccRCC xeno-transplant success rates. In order to better understand the potential relationship between TICs and the immune microenvironment, we employed a multi-modal approach, examining RNA and protein expression (flow cytometry, immunohistochemistry).

**Methods:**

We first examined the gene expression pattern of 18 stem/progenitor marker genes in the cancer genome atlas (TCGA) ccRCC cohort. Flow cytometry was next employed to examine lineage-specific expression levels of stem/progenitor markers and immune population makeup in six, disaggregated, primary ccRCC specimens. Immunohistochemistry was performed on a commercial ccRCC tissue microarray (TMA).

**Results:**

The 18 genes differed with respect to their correlation patterns with one another and to their prognostic significance. By flow cytometry, correlating expression frequency of 12 stem/progenitor markers and CD10 resulted in two clusters—one with CD10 (marker of proximal tubular differentiation), and second cluster containing mostly mesenchymal stem cell (MSC) markers, including CD146. In turn, these clusters differed with respect to their correlation with different CD45^+^ lineage markers and their expression of immune checkpoint pathway proteins. To confirm these findings, four stem/progenitor marker expression patterns were compared with CD4, CD8 and CD20 in a ccRCC TMA which showed a number of similar trends with respect to frequency of the different tumour-infiltrating leukocytes.

**Conclusion:**

Taken together, we observed heterogeneous but patterned expression levels of different stem/progenitor markers. Our results suggest a non-random relationship between their expression patterns with the immune microenvironment populations in ccRCC.

## Background

Renal cell carcinoma (RCC) is a heterogeneous group of carcinomas, of which clear cell carcinoma (ccRCC) comprises over 70% [1]. ccRCC is established to arise from the proximal tubules, and it is marked by frequent expression of CD10, a proximal tubule marker, normally expressed on the brush border of renal tubular epithelial cells [2, 3]. In the kidney, a number of stem/progenitor cells have been identified; these stem/progenitor cells include the multipotent, often perivascular mesenchymal stem cells (MSCs), as well as tubular progenitor cells that appear to be involved in repair of nephrons after renal damage [4–8]. A number of different methods have been employed to isolate these cells, isolating the label-retaining cells, side population cells, as well as sorting by expression of different surface markers [8]. Similarly, stem-like cells (variously labelled cancer stem cells, tumour-initiating cells) have also been isolated from ccRCC specimens using a variety of different surface markers, including CD44, Ecto-5′-nucleotidase (CD73), CXCR4, CD105 and aldehyde dehydrogenase 1 (ALDH1) [9–13]. In turn, expression of these stem/progenitor markers has been associated with generally worse overall survival [14, 15].

Identification of tumour-initiating cells (TICs) may involve functional testing by xenotransplantation limiting dilution assay (LDA). Previously, our group had shown that the microenvironment plays a crucial role in ccRCC xenograft success rates [16]. Using the standard LDA, TICs are calculated to comprise on average 1 in 2 million, and the success rate was greatly enhanced by engrafting small tumour fragments, rather than purified putative TICs. Using this method, as few as 300 cells were sufficient for xenograft success, highlighting the important role for microenvironmental supplementation. While adding back the CD45^+^ cells alone was insufficient to increase engraftment success, the CD45^+^ cells comprised the predominant proportion of viable cells in engrafted tumour specimens.

In patients, the tumour microenvironment (TME) is an important prognostic factor in ccRCC. While greater tumour infiltration by CD8^+^ leukocytes has been associated with better prognosis in a number of cancers [17], ccRCC appears to be an exception, as higher CD8^+^ infiltration is associated with higher grade and worse survival [18, 19]. Extensive CD8^+^ T-cell infiltration, however, comes in different patterns. The tumour-infiltrating lymphocytes (TILs) are found often in forms of cytotoxic T-cells, the activation of which is controlled by coordinated actions of the T-cell receptor (TCR) and the second, co-stimulatory signal via CD28-CD80/86 [20]. Actions of the infiltrating T-cells are dampened by the engagement of various immune checkpoint pathways, including competition of CTLA-4 (CD152) with CD80/86 and the different B7 family members, including PD-L1 (CD274) [20, 21]. When the CD8^+^ T-cell infiltration is accompanied by low expression of immune checkpoints, extensive tumour infiltration was associated with better prognosis [19]. Similarly, expression of PD-1 by the TILs or expression of PD-L1 by the tumour cells are both associated with worse prognosis in ccRCC [22, 23]. In turn, immunotherapy in forms of PD-1/PD-L1 inhibition appears to be useful in ccRCC management, and monoclonal antibodies targeting PD-1/PD-L1 may be combined with tyrosine kinase inhibitors (TKIs) and CTLA4 inhibitors [24, 25].

The prognostic role of the TME is likely to be even more granular—in a recent study of the ccRCC immune microenvironment we identified 17 tumour-associated macrophage phenotypes and 22 T-cell phenotypes [26], where a distinct immune composition correlated with patient survival. Multiple factors are likely playing different roles in shaping the immune TME in ccRCC. Examining gene expression and protein expression, we confirmed heterogeneous phenotypic resemblance to different stem/progenitor cells. In turn, these patterns correlated with different makeup of immune TME, as well as expression patterns of immune checkpoint proteins.

## Methods

### Flow cytometry

Six ccRCC specimens were obtained from the University Health Network (UHN) Program in Biospecimen Sciences from consenting patients in accordance with the policies of the UHN Research Ethics Board (REB ID# 09–0828). Flow cytometry was performed as previously described [27]. Briefly, primary ccRCC specimens were disaggregated into single cell suspensions using collagenase treatment. The specimens were stained using CD45-APC-Cy7 (1:200), CD31-PE-Cy7 (1:200), CD34-PerCP-Cy5.5 (1:50) (all BioLegend) and 1.2 mg/ml TE7-biotin (in-house production from hybridoma obtained from ATCC) followed by Streptavidin-eFluor450 (1:400; eBioscience). These cells were then stained as described in Gedye et al. with an antibody panel that included other stem/progenitor and immune markers [27]. Data collection was performed on a Becton-Dickinson LSR II flow cytometer.

### Tissue microarray and immunohistochemistry

Commercially available tissue microarrays (TMAs) were purchased from US BioMax (HKid-CRCC060PG-01 specs), which contains 60 human cores (30 ccRCC and 30 matched normal kidney). Immunohistochemistry (IHC) conditions for the four stem/progenitor markers are summarized in Table 1, including the pre-treatment conditions. Immunohistochemistry was performed using the standard streptavidin-biotin complex technique, after microwave antigen retrieval on 4-μm sections of formalin-fixed, paraffin wax-embedded tissue.

**Table 1:** Immunohistochemistry conditions

Target	Antibody (Catalogue)	Pre-treatment	Dilution	Incubation
CD44	AbCam (ab157107)	no pretreatment	1/500	1 h
CD90	AbCam (133350)	Citrate pH 6.0	1/300	1 h
CD146 (MCAM)	AbCam (75769)	Tris-EDTA pH 9.0	1/800	1 h
ST3GAL2	Novus (NBPI87044)	Citrate pH 6.0	1/100	Overnight

For the four stem/progenitor markers, weak intra-lesional staining involving < 1/3 of the carcinoma cells was scored as “+”. “+++” score was reserved for moderate to strong staining of > 2/3 of the lesional cells. “++” cores included those with diffuse (> 2/3), weak staining, and those with moderate to strong staining limited to < 1/3 of carcinoma cells. For the immune markers, only strong, membranous staining of CD4, CD8 and CD20 in small round cells were scored as positive staining, and the numbers of stained cells were counted for each core. Any intra-vascular cells or haemorrhagic areas were excluded from the counting.

### Clustering of mRNA levels and flow cytometry results and statistics

The expression levels of stem/progenitor marker genes and immune infiltrate-related genes were downloaded from cBioPortal (http://www.cbioportal.org/), restricted to RNA Seq V2 RSEM z-score values. The data downloaded were limited to the cases published in the 2013 TCGA study, comprised of 417 cases examined by RNA Seq (V2 RSEM) [28]. Unsupervised clustering of mRNA expression and IHC staining patterns were performed using Cluster 3.0, an open-source clustering software, and visualized using TreeView [29].

All statistics were performed using JMP14 (IBM SAS). Comparisons of non-parametric variables (ex. IHC intensity between groups/clusters) were performed using ANOVA. Correlations between two continuous variables were performed using Pearson correlation. Survival analyses were performed using the Kaplan-Meier method.

## Results

### mRNA expression patterns in the TCGA ccRCC cohort

**Fig. 1:**
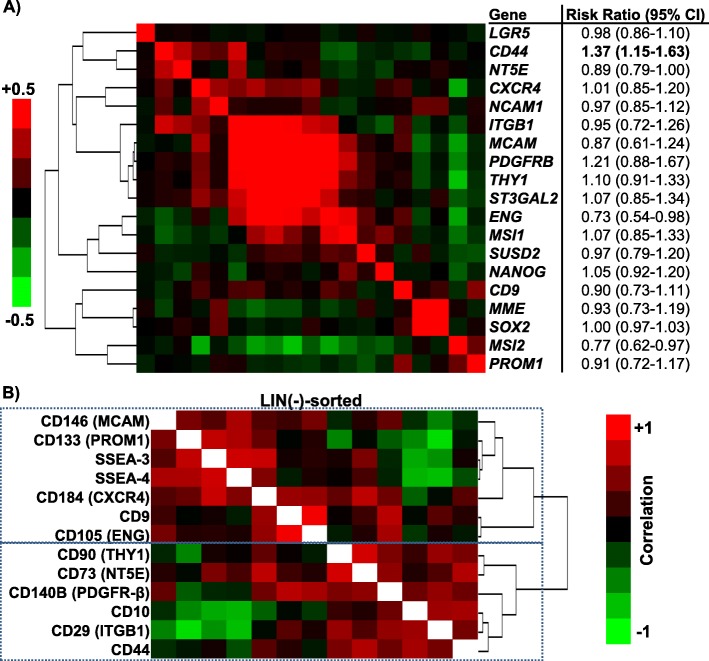
Correlation patterns between the stem/progenitor markers in ccRCC. **a** Clustered correlation, showing the correlation coefficients between mRNA for the different stem/progenitor cell marker genes along with *MME* (encoding CD10), a marker of mature renal tubular cells from the TCGA data. The risk ratios correspond to their impact on the overall survival, examining the gene expression levels (Z-scores) as continuous variables. **b** Clustered correlation heatmap displaying the Pearson correlations between the 12 stem/progenitor cell markers and CD10 in the LIN(−) population

We examined the expression levels of 18 stem/progenitor marker genes, along with *MME*, a marker of mature proximal tubular cells (encoding CD10), in the TCGA ccRCC cohort. Gene expression patterns were heterogeneous in the ccRCC cohort. Examining for correlations between the 19 genes by unsupervised clustering, two distinct clusters were formed (Fig. 1a). The smaller cluster included *CD9*, *SOX2*, *MSI2* and *PROM1* (CD133), along with *MME* (CD10), the marker of mature proximal tubular epithelium. The remaining 14 stem/progenitor marker genes clustered with one another, with particularly strong correlations seen between a number of mesenchymal stem cell (MSC) markers, including *MCAM* (CD146), *PDGFRB* (CD140B), and *THY1* (CD90), with *ST3GAL2*, the rate-limiting enzyme involved in SSEA-3 conversion to SSEA-4.

In the TCGA cohort, these 19 genes were also heterogeneous with respect to their prognostic significance. Among them, *CD44* was the only gene where higher expression was associated with worse overall survival (i.e. higher risk ratio) when the mRNA Z-score was examined as a continuous variable (risk ratio = 1.37, *p* = 0.0005). *CD44* expression correlated negatively with *MME* (Pearson correlation coefficient (PCC) = 0.1242, *p* = 0.0091), suggesting that higher *CD44* mRNA may be associated with either more primitive and/or dedifferentiated carcinomas.

Considering the role for microenvironmental supplementation in tumour xenograft success, we next examined the relationship between the expression levels of the different stem/progenitor marker genes and a set of immune microenvironment-related genes, including a number of T- (*CD2*, *CD5*, *CD7*, *CD4*, *CD8A*), B-cell markers (*CD19*, *MS4A1*, *CD79A*, *CD79B*), as well as a number of immune checkpoint pathway genes (*CD28*, *CD80*, *CD86*, *CD274* (PD-L1), *PDCD1LG2* (PD-L2), *PDCD1* (PD1), *ICOSLG*, *ICOS*, *CD274* and *LAG3*) (Supp. Figure 1). Examining for correlations, *CD44* and *CXCR4* showed particularly strong positive correlations with most of the immune genes examined. *CD276* (B7-H3) expression correlated positively with a number of MSC markers and *ST3GAL2*. While CD276 is frequently expressed on tumour endothelium, CD276 expression has been reported in some tumour cell subsets [30].

While these correlations between stem/progenitor marker gene expression and immune environment makeup, several genes examined, especially *CD44* and *CXCR4*, are expressed by non-tumoural cells. Indeed, *CD44* is expressed by most CD45^+^ cells, and, as expected, *CD44* expression correlated positively with *PTPRC* (CD45) (PCC = 0.2690, *p* < 0.0001).

### Lineage-specific surface marker expression assessment by flow cytometry

Because the above correlations were performed agnostic to lineage specificity (ex. immune vs. carcinoma cells) and overlapping expression by different lineages (esp. *CD44* and *CXCR4*), we next disaggregated primary ccRCC specimens and examined surface expression by flow cytometry. By co-staining with anti-CD45, CD34 and TE7 antibodies [31], we were able to label the different subpopulations as leukocytes (CD45^+^), endothelial cells (CD34^+^), fibroblasts (TE7^+^) and lineage (LIN)-negative cells (i.e. carcinoma cells). Among the 18 stem/progenitor genes examined by mRNA, we examined 12 surface markers, namely CD9, CD29, CD44, CD73, CD90, CD105 (Endoglin), CD133, CD140B, CD146, CD184 (CXCR4), SSEA-3 and SSEA-4, along with CD10. Unsupervised clustering of the 13 surface markers resulted in four discrete clusters that corresponded to labelled lineages (Supp. Figure 2). The 13 surface markers differed with respect to their ability to discriminate between the lineages. For example, CD29 (ITGB1) was expressed at high levels in all the sub-populations, while CD133 (PROM1) was rarely expressed. The LIN-negative sub-population showed the highest frequency of CD10 expression. A number of MSC markers, including CD44, CD73, CD90, CD140B and CD146, were expressed at heterogeneous levels within the LIN-negative sub-population, with higher expression in the fibroblast and endothelial sub-populations. Focusing on the LIN-negative cells, the expression pattern of the 13 surface markers resulted in two correlation clusters, with distinctively different pattern compared to the mRNA-based clusters; one CD10-containing cluster (with CD90, CD73, CD140B, CD29 and CD44), and another cluster containing CD146 (MCAM), along with CD133, SSEA-3, SSEA-4, CD9 and CXCR4 (Fig. 1b).

**Fig. 2:**
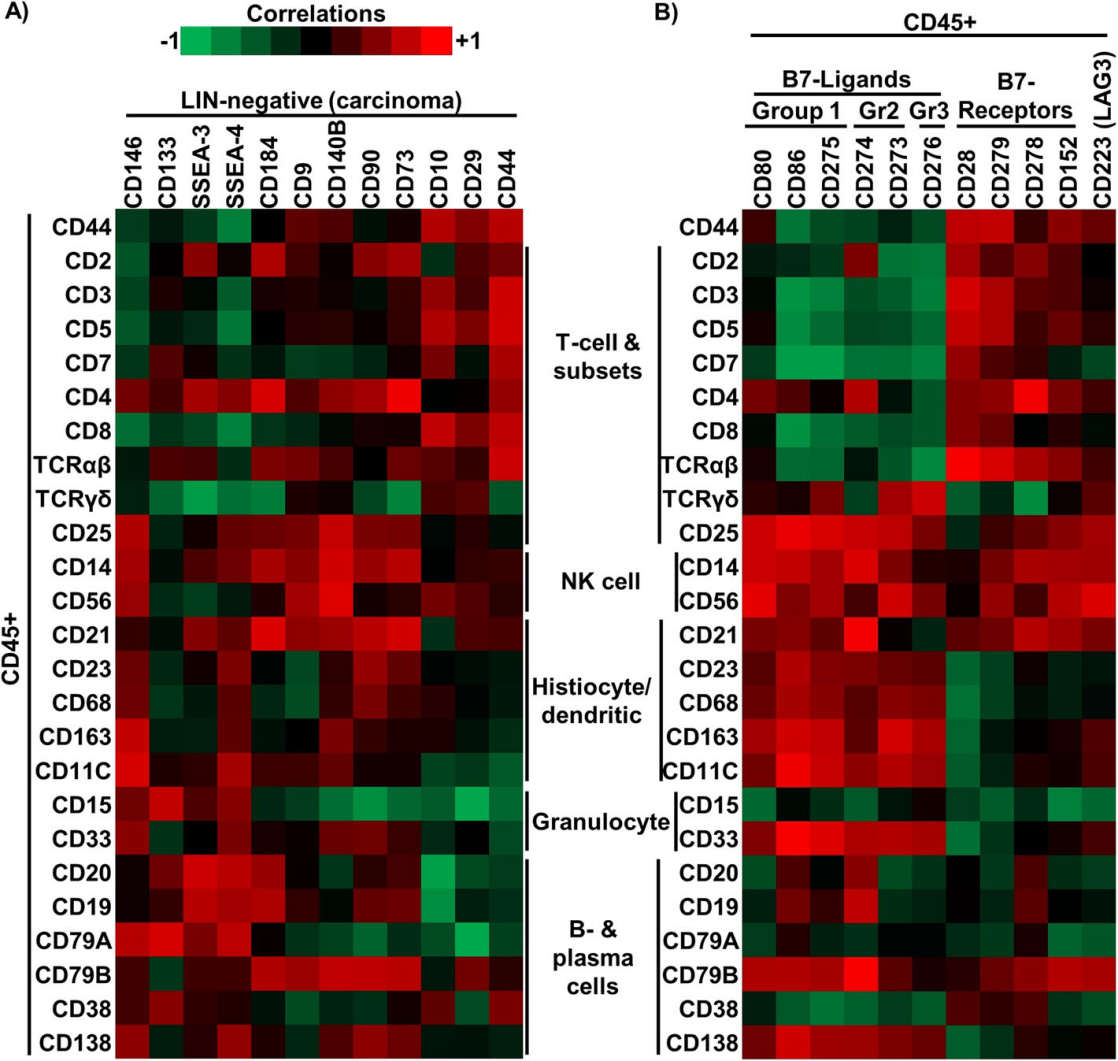
Flow cytometric assessment of the immune microenvironment and its relationship with stem/progenitor marker expression. **a** Correlation heatmap displaying the Pearson correlations between the 13 stem/progenitor cell marker expression levels on the LIN- cells compared to the different CD45+ subset markers. **b** Correlation heatmap displaying the Pearson correlations between the 11 immune checkpoint pathway proteins expression levels on the CD45+ cells compared to the different CD45+ subset markers

We next examined the different sub-populations and compared the expression levels of different immune population markers within the CD45^+^ population with respect to stem/progenitor marker expression in the LIN-negative population (Fig. 2a). Among the stem/progenitor markers, expression of CD10, CD29 and CD44 in the LIN-negative sub-population showed particularly strong positive correlation with higher proportion of CD45^+^ cells expressing a number of T-cell surface markers (i.e. CD3, CD5, CD7, CD8, TCRα/β), suggesting enrichment for cytotoxic CD8^+^ T-cells. In contrast, correlation patterns with SSEA-3 and SSEA-4 suggested enrichment for B-cells (based on correlation with CD19, CD20, CD79A) and CD4^+^ cells.

Next we examined immune checkpoint proteins in the CD45^+^ sub-population (Fig. 2b). B7 family receptors, including CD28 and CD279 (PD-1), generally correlated positively with a number of T-cell markers (Fig. 2 b), likely reflecting the expression of the B7 family receptors by the tumour-infiltrating T-cells. On the other hand, expression of B7 family ligands, including the negative immune checkpoint regulators CD152 (CTLA4), CD274 (PD-L1) and CD273 (PD-L2), correlated positively with NK cell markers (CD14 and CD56), along with histiocytic/dendritic markers (CD21, CD23, CD68, CD163, CD11c), and CD25 (expressed Tregs, among others).

**Fig. 3:**
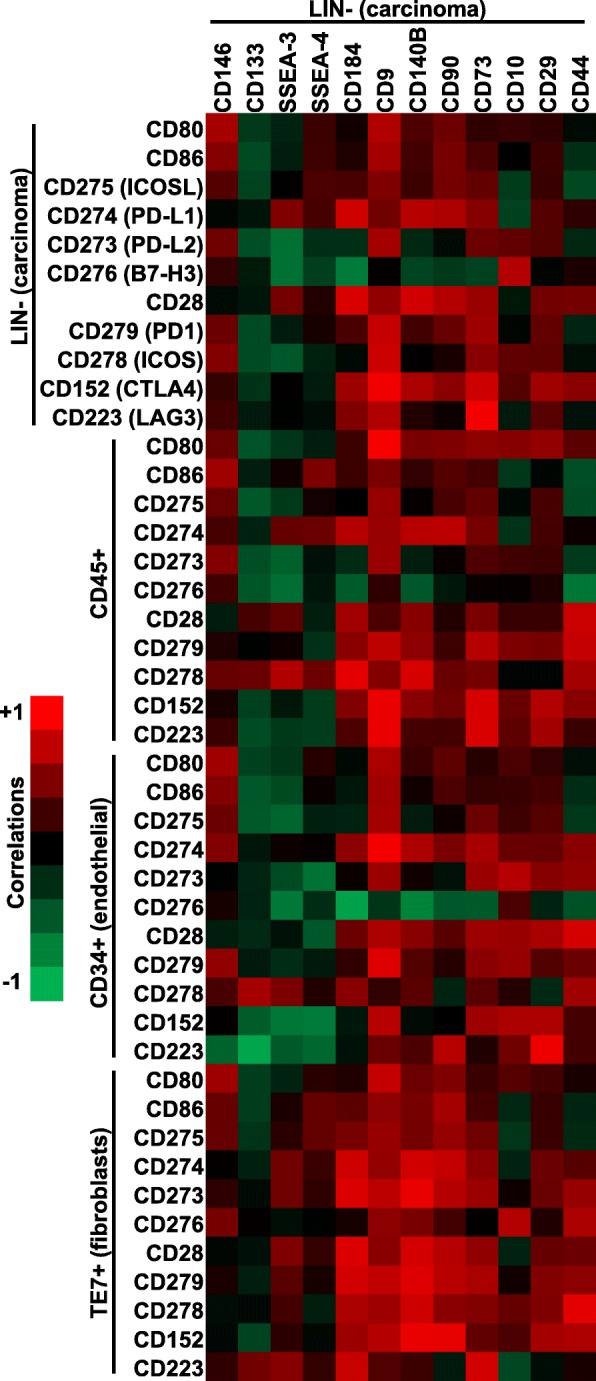
Correlation heatmap displaying the Pearson correlations between the 13 stem/progenitor cell marker expression levels on the LIN- cells compared to the different immune markers in the four sub-populations

When we examined the expression patterns of the immune checkpoint proteins on different sub-populations, CD140B and CD90 positivity in the LIN-negative sub-population showed generally positive correlation with the expression of most of the immune checkpoint proteins examined (Fig. 3). These correlations were seen across the different sub-populations, including endothelial cells and fibroblasts. On the other hand, CD133, SSEA/3 and SSEA-4 correlated negatively with most of the immune checkpoint proteins examined, and these correlations were more striking in the CD45^+^ and CD34^+^ sub-populations. Taken together, these correlation patterns point to a novel relationship between the immune microenvironment with different patterns of stem/progenitor marker expression by carcinoma cells.

### Immunohistochemical examination of stem/progenitor markers and the immune microenvironment

**Fig. 4:**
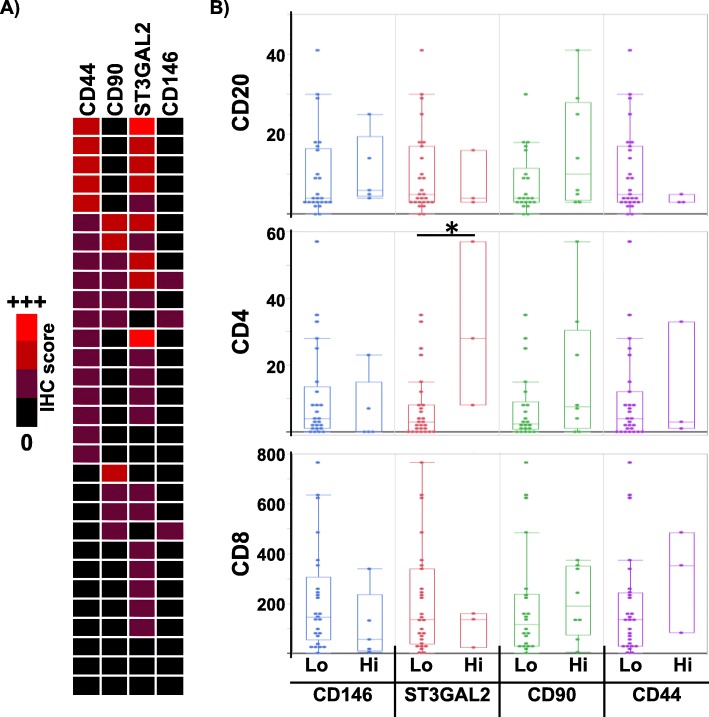
Tissue microarray results. **a** Graphical summary of the IHC results from 30 ccRCC cores, with the indicated staining intensity. **b** Frequency of the indicated immune infiltrate in cores with carcinomas expressing the indicated stem/progenitor marker proteins. * indicates Wilcoxon test *p* = 0.0282. Other unmarked comparisons were non-significant (*p* > 0.05)

In order to confirm our flow cytometry-based observations, we examined four stem/progenitor markers (CD44, CD90, CD146, ST3GAL2) and three immune markers (CD4, CD8, CD20) by immunohistochemistry on tissue microarray (TMA) sections, comprised of 60 cores (30 ccRCCs and 30 matched normal kidney) (representative cores in Supp. Figure 3 and graphical summary in Fig. 4a). In the normal kidney cores, strong CD146 staining is seen also in the vessel walls, including the glomerular arterioles. This vascular staining pattern is retained in most ccRCC cores, highlighting the delicate intra-tumoural vessels. Some cores showed membranous staining of individual and small groups of tumour cells (up to 2+ staining) in 5/30 cases, and this pattern was interpreted as true lesional staining.

In the normal kidney cores, CD90 expression is seen in some vessel walls and the renal tubules, more predominantly in the proximal portions, with apical accentuation, likely corresponding to the brush borders (Supp. Figure 4). CD90 staining in vessel walls could be seen in rare cases. In ccRCC cases, CD90 staining is mostly in the vessel walls, with some larger cells being highlighted, perhaps representing fibroblasts. Membranous staining of the carcinoma cells, either as individual or small groups of cells was observed in a subset, with relatively diffuse staining seen in 8/30 cases.

Patchy, cytoplasmic ST3GAL2 expression is seen in the non-tumoural glomeruli and the vessel walls. In ccRCC cases, 10/30 cases showed lesional, cytoplasmic ST3GAL2 staining. Negative cases showed weak staining in the vessel walls, without discernible lesional staining. CD44 expression in the normal kidney cores was seen in some vessel walls and small mononuclear cells, interpreted to represent leukocytes. Only weak CD44 expression was seen in 3/30 (10%) of the tumours. For the other cores, CD44 stained a number of smaller, intra-lesional cells, interpreted to represent TILs, and negative in the actual tumour cells, with some vascular staining.

There was no clear pattern with respect to tumour grade and stem/progenitor marker staining; 22/30 cases were of nucleolar grade 2, and most positively stained cases were of grade 2. Comparing the different stem/progenitor markers, the cores with stronger CD90 staining tended to show stronger CD146 staining (Fig. 4a). Otherwise, there were no clear patterns with respect to the relationship between the different markers.

We next examined the same TMA for frequency of CD4^+^, CD8^+^ and CD20^+^ cells (Supp. Figure 4). The number of CD8^+^ cells was not significantly different based on the stem/progenitor marker expression, although the number of CD8^+^ cells tended to be lower in CD146^High^ and ST3GAL2^High^ cores, in line with the flow cytometry- and the mRNA-based observations (Fig. 4b). CD4^+^ cells were more frequent with ST3GAL2^High^cores (Wilcoxon *p* = 0.0282). CD20^+^ cells also tended to be more frequent in CD90^High^ cores. While most comparisons did not reach statistical significance, the trends were similar to that seen with the flow cytometry data.

Taken together, our results confirm that the immune microenvironment in ccRCC is heterogeneous with respect to both its makeup and immune checkpoint protein expression. There is also striking heterogeneity in the expression patterns of different stem/progenitor markers. Correlations and clustering patterns seen between these features suggest a non-random relationship, the significance of which remains to be elucidated.

## Discussion

Adult tissue regeneration is a dynamic, homeostatic process that engages a number of different stem/progenitor cells. This may also be true in cancer growth, where different subtypes of TICs, with heterogeneous surface marker profiles, may be engaged in cancer growth in patients and in cancer reconstitution in xenograft models. Examining the different possible stem/progenitor markers in ccRCC, we observed different patterns of correlation clusters. These patterns showed heterogeneity across different modalities utilized. Part of the discrepancy is related to their expression by different lineages (ex. *CD44*), an issue that was addressed by examining lineage-specific expression by flow cytometry. Flow cytometry also allowed for robust distinction between the different infiltrating leukocytes. However, distinction between true TILs and bystander leukocytes is perhaps best done by IHC, especially considering the rich vasculature and frequent haemorrhage that can be seen in ccRCC specimens. The clear drawback in IHC was encountered in assessing CD90 and CD146 staining, where distinguishing between vascular and true lesional staining was difficult.

Despite these challenges, some of the patterns seen from the TMA IHC data showed similar trends seen with flow cytometry data, with both data sets pointing to a non-random relationship between stem/progenitor marker expression and the immune microenvironment makeup. A subset of stem/progenitor markers correlated positively with CD8^+^ T-cell markers (ex. CD10, CD44), while others correlated positively with B-cell markers (ex. SSEA-3/SSEA-4). Interestingly, a different set of stem/progenitor markers showed the strongest positive correlations with the different immune checkpoint inhibitor proteins (ex. CD140B), including CD274 (PD-L1). This is an interesting biological discordance that may have treatment implications in immune checkpoint therapy.

An interesting question is regarding the stem/progenitor cell marker expression significance. Expression of different stem/progenitor marker genes and proteins may be an acquired phenotype in the setting of epithelial-mesenchymal transition (EMT). ccRCC is a peculiar carcinoma, being one of the few carcinomas that normally express both cytokeratin and vimentin type intermediate filaments [32, 33], the latter being a well-established marker of mesenchymal differentiation [34]. When aberrantly expressed in carcinoma cells, vimentin is seen as a marker of EMT. However, electron microscopic features of ccRCC are that of carcinoma, with long microvilli on the apical surface and numerous cell junctions [35], with the microvilli being the highlighted by (apical) CD10 staining [2, 3]. Dedifferentiation, perhaps related to EMT, can be seen in ccRCC. This is seen in the form of sarcomatoid (de-)differentiation, marked by spindle cell histology, which may be accompanied by CD10 loss [36]. Increased CD44 expression has been associated with sarcomatoid differentiation and aberrant p53 expression in renal cell carcinoma [37–39]. Sarcomatoid differentiation has been shown to harbour significantly higher mutational burden [39], along with higher PD-1 and PD-L1 (CD274) expression [40]. Unfortunately, the number of tumours cores with sarcomatoid foci was too small for a meaningful comparison. Regardless, there lacked a clear relationship between tumour grade with TIL frequency and stem/progenitor marker expression patterns, suggesting that EMT is unlikely to be the main biological driver.

A number of stem/progenitor markers examined in this study have been used to isolate TICs/cancer stem cells in ccRCC and many other cancers. While tempting, higher expression of these markers cannot be equated with TIC enrichment. In particular, CD90 may be a particularly poor marker; CD90 staining pattern in the normal proximal tubules was reminiscent of that seen with CD10, raising the possibility that CD90 expression may simply be a retained phenotype. For others, while a number of MSC markers have been used to isolate TICs in many settings, quantifying TICs is best performed by functional assays, as we had previously performed [16]. Unfortunately, because these functional assays are generally performed in systems lacking a functional immune system, the impact of TICs on the immune microenvironment remains poorly understood. Could the other stem/progenitor marker expression be an intrinsic (*vs*. acquired) feature in some carcinomas, related to their cell-of-origin? While ccRCC is generally well established to arise from the proximal tubules, a number of different stem/progenitor cells are well-capable of regenerating those structures and may serve as potential cells-of-origin for a subset of ccRCC. It is possible that a subset of ccRCCs may be more primitive than others, a feature not derived through dedifferentiation but rather related to their origin. While this question is outside of the scope of this study, the possibility is raised, in which case the carcinoma-immune microenvironment relationship may be a feature established early in tumourigenesis.

A number of interesting correlations have been uncovered in this study, but this study was limited to correlations. We were also limited by a much smaller sample size for flow cytometric analysis, thus limiting the power of some of the correlations observed. However, our results point to a novel, unexplored relationship between cancer pathogenesis and the immune microenvironment. Going forward, a more solid conclusion about the relationship between the nature of the TICs and the microenvironment may be attainable by serially following and comparing the tumour and microenvironment makeup.

## Conclusions

The importance of the immune microenvironment in ccRCC is obvious, with respect to both prognosis and therapy. Understanding the marked heterogeneity in the immune microenvironment will be pivotal in choosing the right patients for immune checkpoint therapy. Tumour mutational burden and presentation of neo-antigens through the immunoproteasome pathway is one of the factors that shape the microenvironment; our data highlight immunophenotypic resemblance of ccRCC to different stem/progenitor cells as another potential factor. Further studies are required to verify this relationship and to understand its significance.

## Supplementary information

**Additional file 1: Supp. Figure 1.** Correlation heatmap displaying the Pearson correlations between the 19 stem/progenitor cell marker genes in the TCGA cohort *vs*. *PTPRC* and immune checkpoint inhibitor genes.

**Additional file 2: Supp. Figure 2.** Clustered expression heatmap, displaying the expression levels for 13 markers (12 stem/progenitor cell markers and CD10) in the six patient samples, in the four different cell populations as indicated.

**Additional file 3: Supp. Figure 3.** A) Representative immunostaining results from normal (left-most column) and ccRCC cores (centre and right-most columns). Bar = 100 μm.

**Additional file 4: Supp. Figure 4.** Representative immunostaining results for cores with high CD4, CD8 or CD20 counts. Bar = 100 μm.

## Data Availability

The datasets used and/or analysed during the current study are available from the corresponding author on reasonable request.

## Abbreviations

ccRCC: Clear cell renal cell carcinoma
IHC: immunohistochemistry
LDA: Limiting dilution assay
MSC: Mesenchymal stem cell
RCC: Renal cell carcinoma
TCR: T-cell receptor
TCGA: The cancer genome atlas
TMAs: Tissue microarrays
TICs: Tumour-initiating cells
TILs: Tumour-infiltrating lymphocytes
TME: tumour microenvironment
