# Supplementary material for: Stem/progenitor cell marker expression in clear cell renal cell carcinoma: a potential relationship with the immune microenvironment to be explored
Source: BMC Cancer. 2020 Apr 3;20:272. doi: 10.1186/s12885-020-06733-4 (PMC7119074; doi:10.1186/s12885-020-06733-4)
Supplement: Supplementary file 1 [file 12885_2020_6733_MOESM1_ESM.docx]

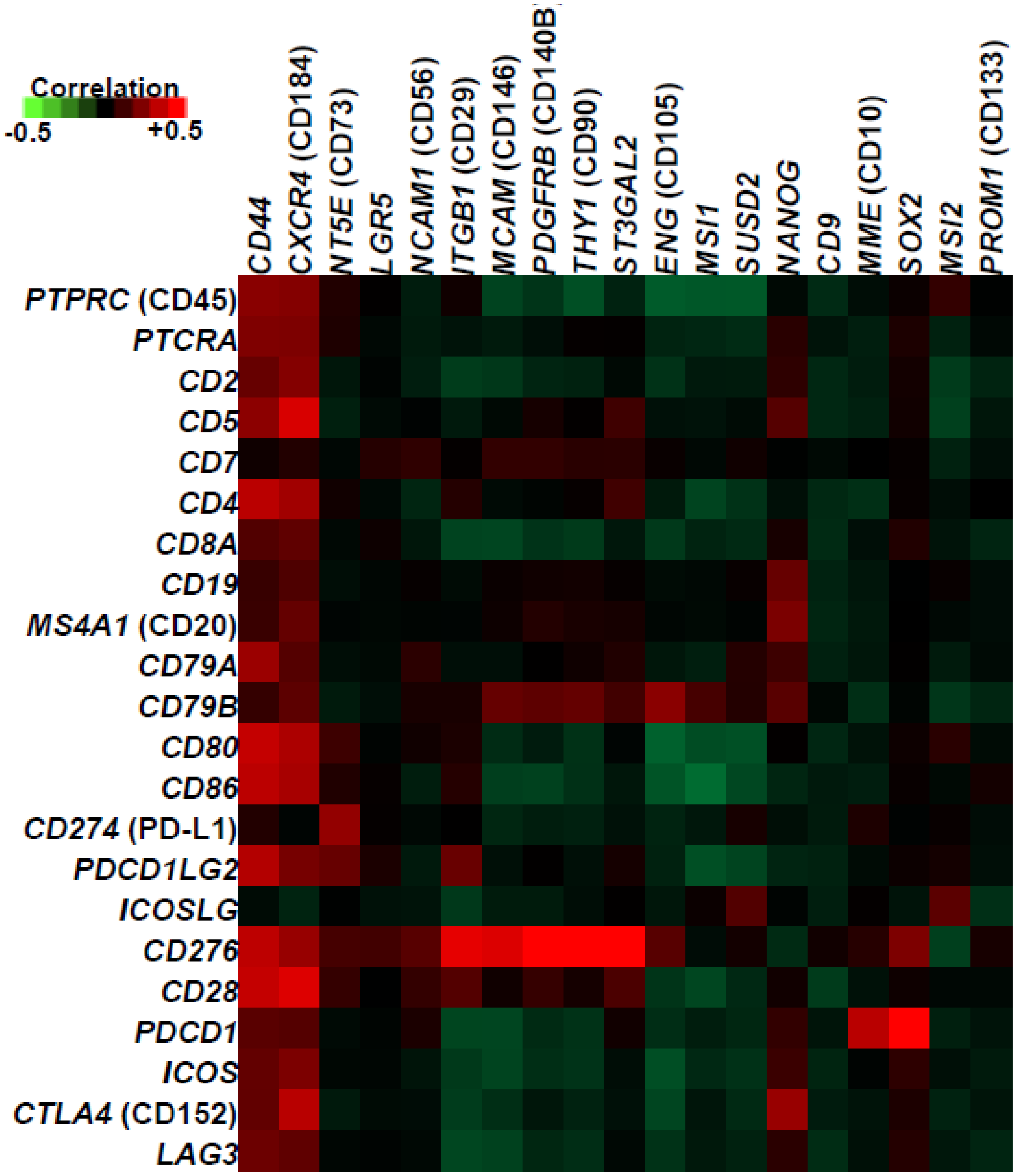


**Supp. Fig. 1.** Correlation heatmap displaying the Pearson correlations between the 19 stem/progenitor cell marker genes in the TCGA cohort *vs. PTPRC* and immune checkpoint inhibitor genes.
