# Supplementary material for: Stem/progenitor cell marker expression in clear cell renal cell carcinoma: a potential relationship with the immune microenvironment to be explored
Source: BMC Cancer. 2020 Apr 3;20:272. doi: 10.1186/s12885-020-06733-4 (PMC7119074; doi:10.1186/s12885-020-06733-4)
Supplement: Supplementary file 2 [file 12885_2020_6733_MOESM2_ESM.docx]

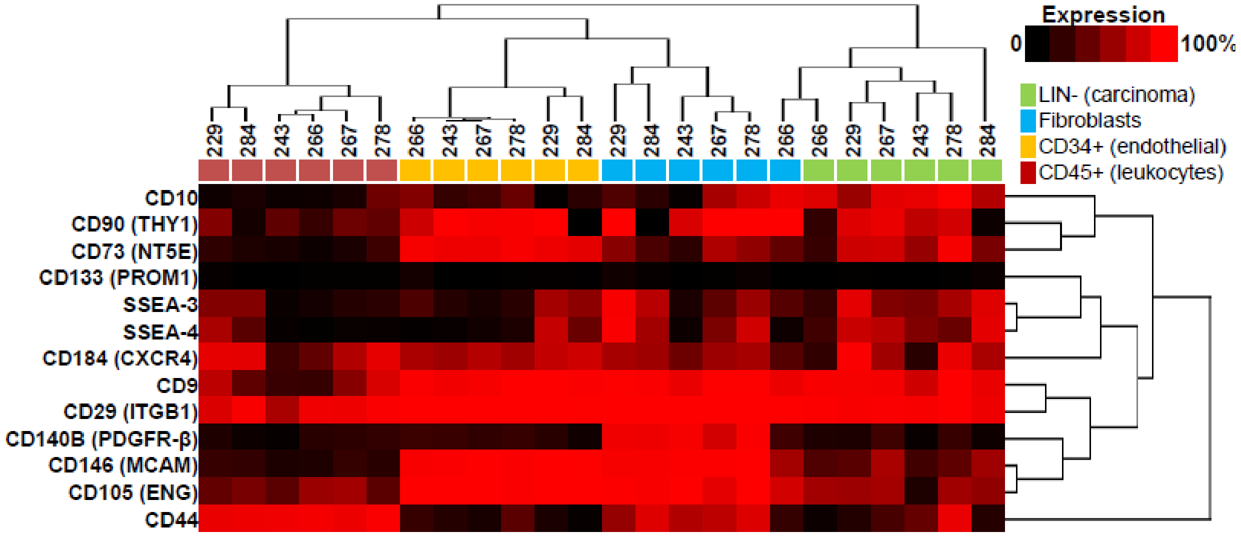

Supp. Fig. 2. Clustered expression heatmap, displaying the expression levels for 13 markers (12 stem/progenitor cell markers and CD10) in the six patient samples, in the four different cell populations as indicated.
