# Supplementary material for: Stem/progenitor cell marker expression in clear cell renal cell carcinoma: a potential relationship with the immune microenvironment to be explored
Source: BMC Cancer. 2020 Apr 3;20:272. doi: 10.1186/s12885-020-06733-4 (PMC7119074; doi:10.1186/s12885-020-06733-4)
Supplement: Supplementary file 3 [file 12885_2020_6733_MOESM3_ESM.docx]

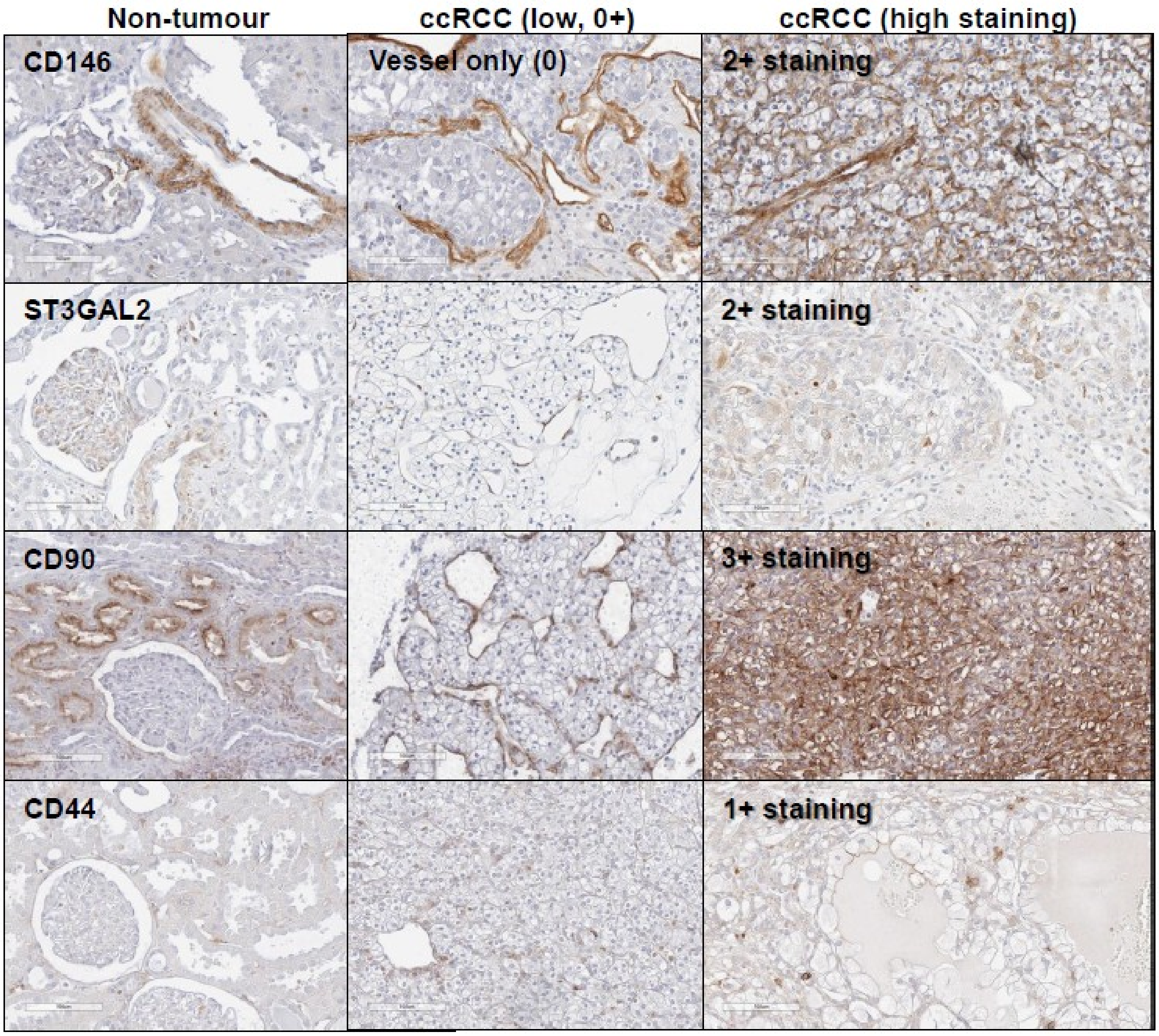

Supp. Fig. 3. Representative immunostaining results from normal (left-most column) and ccRCC cores (centre and right-most columns). Bar = 100 µm.
